# Spatial patterns of incident malaria cases and their household contacts in a single clinic catchment area of Chongwe District, Zambia

**DOI:** 10.1186/s12936-015-0793-1

**Published:** 2015-08-07

**Authors:** Jessie Pinchoff, German Henostroza, Bryan S Carter, Sarah T Roberts, Sisa Hatwiinda, Busiku Hamainza, Moonga Hawela, Frank C Curriero

**Affiliations:** Department of Epidemiology, Johns Hopkins University Bloomberg School of Public Health, 615 N Wolfe St, Baltimore, MD 21205 USA; Centre for Infectious Disease Research Zambia, 5032 Great North Road, Lusaka, Zambia; Icahn School of Medicine at Mt Sinai, 1428 Madison Avenue, New York, NY 10029 USA; Department of Epidemiology, University of Washington School of Public Health, Box 357236, Seattle, WA 98165 USA; Ministry of Health, National Malaria Control Centre, Chainama Hospital, College Grounds, Off Great East Road, PO Box 32509, Lusaka, Zambia; University of Alabama at Birmingham, 1900 University Boulevard, Birmingham, AL 35294 USA

**Keywords:** Malaria, Spatial statistics, Surveillance, Active case detection

## Abstract

**Background:**

Reactive case detection (RACD) for malaria is a strategy that may be used to complement passive surveillance, as passive surveillance fails to identify infections that are asymptomatic or do not seek care. The spatial and seasonal patterns of incident (index) cases reported at a single clinic in Chongwe District were explored.

**Methods:**

A RACD strategy was implemented from June 2012 to June 2013 in a single catchment area in Chongwe District. Incident (index) cases recorded at the clinic were followed up at their household, and all household contacts were tested for malaria using rapid diagnostic tests (RDTs). GPS coordinates were taken at each index household. Spatial analyses were conducted to assess characteristics related to clustering, cluster detection and spatial variation in risk of index houses. Effects of season (rainy versus dry), distance to the clinic and distance to the main road were considered as modifying factors. Lastly, logistic regression was used to identify factors associated with the proportion of household contacts testing RDT positive.

**Results:**

A total of 426 index households were enrolled, with 1,621 household contacts (45% RDT positive). Two space–time clusters were identified in the rainy season, with ten times and six times higher risk than expected. Significantly increased spatial clustering of index households was found in the rainy season as compared to the dry season (based on K-function methodology). However, no seasonal difference in mapped spatial intensity of index households was identified. Logistic regression analysis identified two main factors associated with a higher proportion of RDT positive household contacts. There was a 41% increased odds of RDT positive household contacts in households where the index case was under 5 years of age [OR = 1.41, 95% confidence intervals (1.15, 1.73)]. For every 500-m increase in distance from the road, there was a 5% increased odds of RDT positive household contacts [OR = 1.05 (1.02, 1.07)], controlling for season.

**Discussion:**

Areas of increased report of malaria persist after controlling for distance to the clinic and main road. Clinic-based interventions will miss asymptomatic, non-care seeking infections located farther from the road. RACD may identify additional infections missed at the clinic.

## Background

The World Health Organization estimates that there were 207 million cases of malaria globally in 2012 with 627,000 deaths [1]. Transmission in Zambia has been declining; the 2012 malaria indicator survey found a malaria parasite prevalence of 14.9% nationally down from 16% in 2010 [2]. Since the late 1990s, Zambia has scaled up malaria control activities, including the distribution of long-lasting, insecticide-treated nets (LLINs), indoor residual spraying, prompt diagnosis and treatment with artemisinin combination therapy (ACT) and intermittent preventive therapy in pregnant women [3]. The surveillance system in Zambia, as in most moderate and high transmission settings, is based on passive case detection, in which symptomatic individuals seek care at the clinic or hospital. As transmission declines, it becomes critical to enhance existing surveillance methods, as many infections are asymptomatic or have low access to healthcare facilities and do not receive treatment. Reactive case detection (RACD) is one form of active case detection in which all individuals in close proximity to a passively detected ‘index’ case are tested and treated, including if they are asymptomatic [4–6].

RACD is based on the assumption that infections are clustered in space and time, within transmission ‘hotspots’ [5, 7, 8]. In Zambia, a study found that the prevalence of malaria infection was significantly higher within passively detected index case households as compared to randomly selected control households (8 vs 0.7%, respectively) [4]. To maximize the impact and cost effectiveness of RACD, the strategy should be targeted based on geographic or demographic risk factors [9].

Malaria transmission is heterogeneous at varying geographical scales, and transmission can vary significantly by season. This heterogeneity is driven by a variety of ecological, biological and sociological factors. Passive surveillance may only test and treat the sub-set of infections that seek care at the facility. Describing the spatial temporal distribution of passively detected households may help to better understand care seeking and malaria transmission dynamics in this peri-urban setting.

The aims of this study were to describe characteristics of the spatial distribution of passively detected index households for malaria as identified at the clinic. Tools from the field of spatial statistics were used to assess clustering, cluster detection and spatial variation in risk for incident malaria cases. Seasonal effects were also considered. Lastly, the composition of index households was explored, to describe the rapid diagnostic test (RDT) positivity among household contacts, as related to the households’ distance from the clinic.

## Methods

### Study site and procedures

The study site is located in Chongwe District about 35 km from Lusaka, the capital city of Zambia. The area is considered rural to peri-urban with families living in homesteads and relying on subsistence farming. The clinic catchment area is estimated to be 14,000 individuals. A major paved road runs through the centre of the catchment area. The clinic is near the paved road about in the centre of the catchment area. A swampy area created by a natural hot spring and likely a mosquito-breeding site resides in close proximity to the clinic.

In Zambia, the single rainy season lasts from November through April, followed by a cool season from April until August, and a hot dry season through November; malaria transmission peaks during the rainy season [10]. The primary vectors for malaria in Lusaka Province are *Anopheles gambiae*, *Anopheles funestus* and *Anopheles arabiensis* [11]. Malaria transmission in the area has been declining; Lusaka Province recorded the lowest parasitaemia in the country in 2012 [12].

Cases were passively detected at the clinic between June 2012 and June 2013. The study procedures involved testing each febrile individual seeking diagnosis and treatment at the clinic with a RDT and/or microscopy (Giemsa thick smear) to confirm malaria infection. Those that tested positive were approached for enrolment. RDT positive patients were randomly selected for study participation. Those that were approached and gave informed consent were enrolled in the study and administered a questionnaire. If a selected patient declined to participate, then a randomly selected back-up was approached. Demographic characteristics and malaria symptoms and treatment history were collected and household GPS coordinates recorded. A household contact questionnaire and RDT was administered at the household. Data were obtained on the total number of other house members positive for malaria infection, of those that were present at the time of the survey. All participants who tested positive for malaria in the clinic or during household visits were treated according to national guidelines.

### Statistical analysis

All structures in the catchment area resembling households were enumerated and digitized to provide latitude and longitude coordinates based on a high-resolution satellite image (QuickBird WorldView, DigitalGlobe Services). This methodology was piloted and applied in a study in Lusaka and found to be highly accurate [13]. Environmental features were digitized from the satellite image and GPS coordinates taken in the field, for features such as the paved road, the swamp area and the clinic location. Data were imported into ArcGIS v10.2 and spatially integrated with data on index households.

A series of analysis were conducted to characterize the spatial distribution of the index cases of malaria infection using tools from the field of spatial statistics to assess cluster detection, spatial variation in risk and spatial clustering. The first analysis conducted was to detect spatial–temporal clusters, sub-areas within the clinic catchment area where the number of malaria index cases over an identified period of time is significantly more than what would have been expected given the distribution of all index cases. The spatial scale for this analysis was aggregated to 500-sq m grid cells overlaid onto the study area accumulating the total number of enumerated households (population) and total number of index cases within each grid cell. The temporal scale was aggregated to a week (7 days). Analysis was performed using the cluster detecting software SaTScan [14] specifying the Poisson model approach to accommodate the count of index cases per grid cell relative to the corresponding grid cell population. Identified space–time clusters were restricted to be no larger than 50% of the total study area population at risk (a maximum of 4-km radius) and comprise no more than 4 weeks of available data. Cluster detection analysis for grid cell index case counts was performed adjusting for each grid cell population as well as controlling for distance to the clinic and distance to the road (distances were summarized into quartiles and calculated using the grid cell centroid location).

The second analysis was conducted to jointly explore spatial clustering of malaria index cases, a different characteristic than cluster detection, and spatial variation in risk of malaria index cases [15]. Spatial clustering of malaria index cases seeks to assess the degree to which cases tend to be near other cases, and not necessarily to the degree to which a cluster is formed, whereas spatial variation in risk explores where these cases are occurring to identify spatial trends in the risk of such an event occurring. Of particular interest was how clustering and spatial variation in risk of malaria index cases changed as a function of season (rainy or dry). The K-function, which estimates the expected number of other cases within a range of distances of an arbitrary case and plots a scaled version of these estimates as a function of distance, was used to assess spatial clustering [15, 16]. Spatial intensity, defined as the expected number of cases per unit area, was estimated in a non-parametric fashion using the kernel density approach and mapped to highlight spatial variation in the concentration of cases [15, 16]. Both K-function analysis and spatial intensity were performed using the R Statistical software spatstat [17].

The final analysis conducted was to identify factors associated with a higher proportion of household contacts that were RDT positive. Knowing the total number of household members (contacts) per index house and the number of other household members testing positive, logistic regression was used to model the proportion of household contacts positive for malaria. Variables explored in the regression include distance to the paved road, distance to the clinic, as well as age and gender of the index case.

This study received ethical approval from both the University of Zambia Research Ethics Committee and the University of Alabama at Birmingham Institutional Review Board.

## Results

A total of 5,383 individuals were tested between June 2012 and June 2013; 3,061 tested positive for malaria and 426 (14%) enrolled as index cases. Within the 426 index households, 1,621 household contacts were tested, of which 735 (45%) were RDT positive. An additional 2,546 structures were enumerated based on satellite imagery. Index households were located closer to the clinic, swamp and main road, as compared to enumerated structures: the median distance to the clinic for index households was 9.2 km compared to 15 km for all other enumerated households; the median distance to the swamp for index households was 6.6 km compared to 12.3 km for enumerated households; and the median distance to the road was 4.9 for index households and 8.8 km for enumerated households 8.6 km (Table 1). The median number of RDT positive household contacts within an index household was two individuals, with the total number of household members present during follow-up between none and ten individuals (Table 1). The number of index cases identified at the clinic was higher in the rainy season, and the proportion of RDT positive household residents was over 25% each month, peaking in March with 51% RDT positive.

**Table 1:** Characteristics of households (enumerated) in the clinic catchment area and enrolled households (index) between 2012 and 2013

	Index N (%)	Enumerated N (%)
Number of households	426	2,546
Distance to clinic (km) [median (min, max)]	9.22 (0.23, 27.54)	15 (0.04, 35.86)
Distance to swamp (km) [median (min, max)]	6.61 (0, 25.49)	12.25 (0, 32.60)
Distance to road (km) [median (min, max)]	4.94 (0.32, 24.94)	8.75 (0.01, 26.65)
Index is under 5 years of age	185 (50%)	NA
Index is female	198 (53%)	NA
Visit in rainy season	257 (60%)	NA
Number of total household contacts in index household [median (min, max)]	5 (0, 13)	NA
Number of RDT+ household contacts in index household [median (min, max)]	2 (0, 10)	NA

*NA* not applicable.

**Fig. 1:**
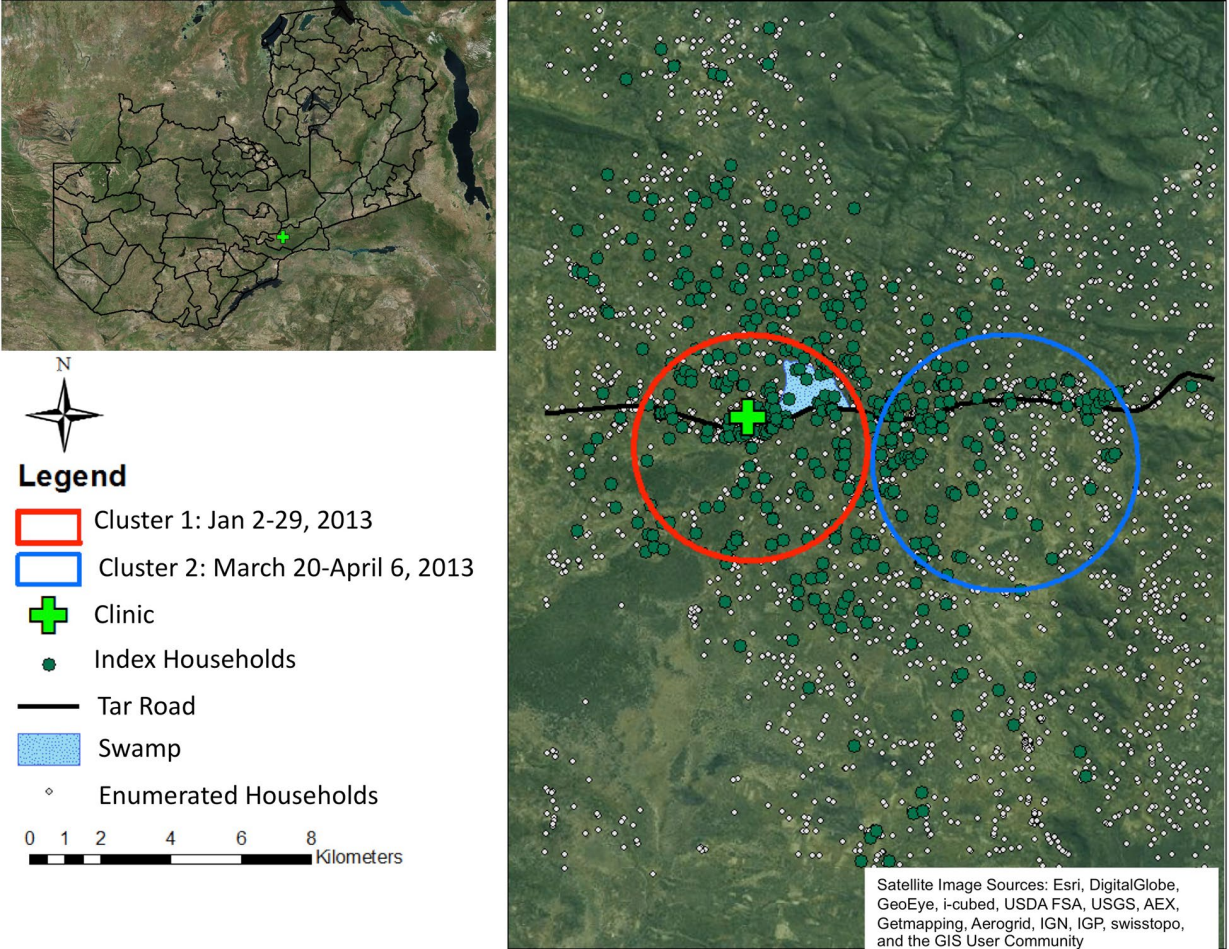
Mapped index houses, enumerated households and identified space–time clusters of incident index malaria households in the clinic catchment area, 2012–2013. Map identifies two space–time clusters of index cases detected, one between 2 and 29 January, 2013 and one between 20 March 20 and 6 April, 2013.

The cluster detection analysis identified two significant clusters of index cases of malaria (Fig. 1). The first cluster was identified in the 4 weeks of January 2013 (2–29 January), with an estimated relative risk of 9.7, a approximately ten-fold increase in the observed index cases than would have been expected under the Poisson assumption of space–time constant risk. The second cluster was identified in the four-week span from 20 March to 16 April, 2013, with an estimated relative risk of 4.2, approximately four times more cases than would have been expected. Both identified clusters, which are shown in Fig. 1 as a circular radius encompassing the included grid cells, persisted after controlling for distance to the clinic and the road.

**Fig. 2:**
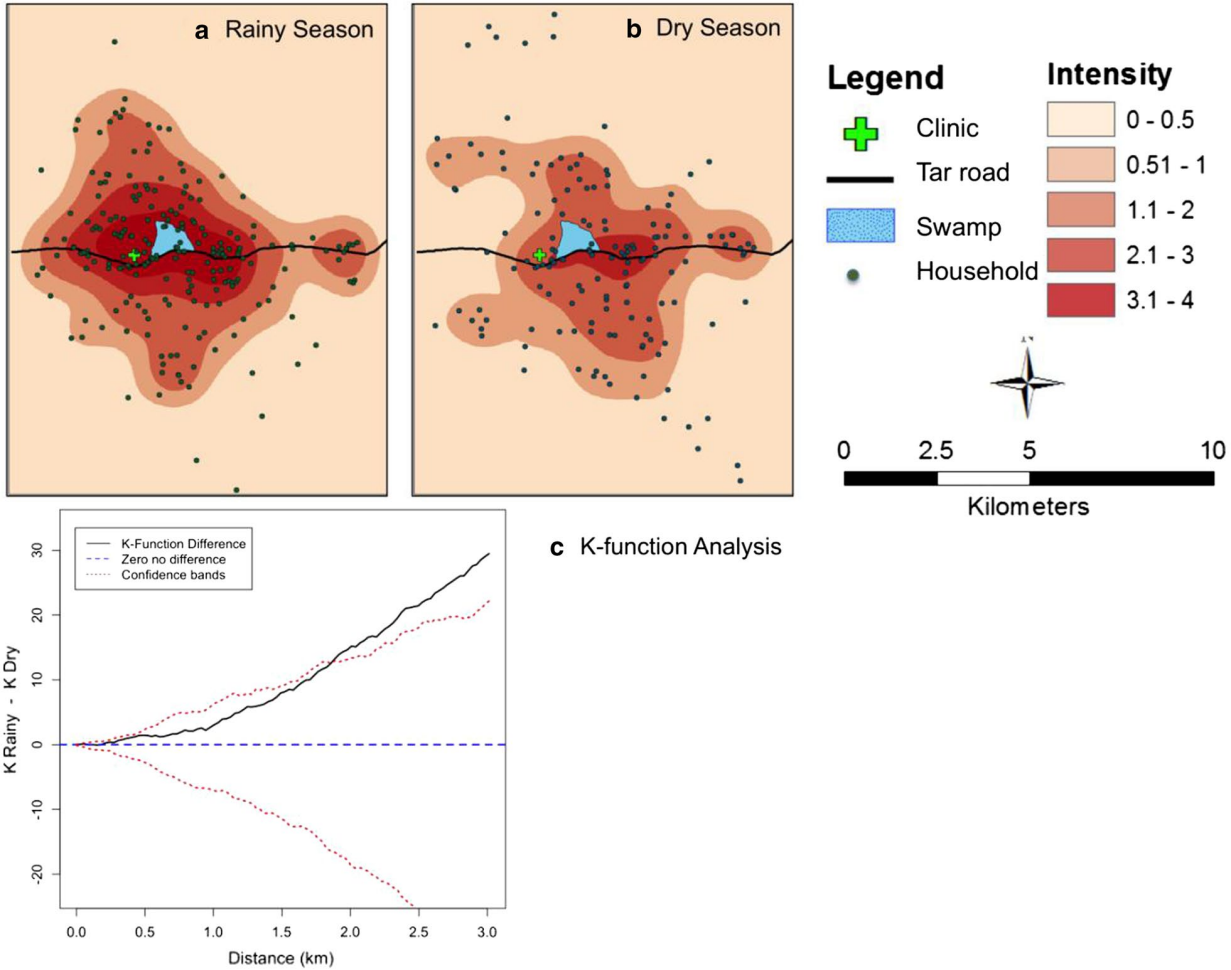
Estimated spatial intensity for index households in the rainy season (**a**) and dry season (**b**). Also shown is the K function difference comparing spatial clustering in the rainy versus dry season (**c**). Figure has three panels that display the estimated spatial intensity for index households calculated in the rainy season (**a**) and dry season (**b**). The third panel is the K function difference comparing the degree of spatial clustering by season (**c**).

Figure 2 displays the results from the clustering and spatial variation in risk of index malaria cases focused on exploring changes across the rainy and dry seasons. Panels A and B of Fig. 2 display spatial intensity, the expected number of index malaria cases per unit area, for both the rainy and dry season case data, respectively. Spatial patterns in concentration of index cases appear relatively consistent across the two seasons with both indicating upwards of three to four index cases expected per sq km in areas focused around the clinic and swamp. Panel C plots the difference in K-functions, K-function for the rainy seasons minus the K-function for the dry season. The result suggests clustering of cases in the rainy season to be more than in the dry season (cases in the rainy season are spatially more compact) since this difference is consistently above the no difference zero line. This difference in clustering becomes statistically significant after about 1.75 km. Confidence envelopes shown in this plot were generated using the random labelling approach [16].

Logistic regression was performed to determine factors associated with having a higher proportion of RDT positive household contacts. In univariate models, the odds of having a higher proportion of RDT positive household contacts increased when the index case was under 5 years of age [OR = 1.42, 95% confidence interval (1.17, 1.73)], and the odds increased per 500-m increase in distance from the clinic [OR = 1.03 (1.01, 1.05)] and from the road [OR = 1.05 (1.03, 1.07)] (Table 2). In a fully adjusted model, there were 41% higher odds of having a higher proportion of RDT positive household contacts in households where the index case was under 5 years of age [OR = 1.41 (1.15, 173)] (Table 2). As distance to the road increased by 500 m, the odds of having a higher proportion of RDT positive household contacts increased 5% [OR = 1.05 (1.02, 1.07)] (Table 2). Model evaluation based on the Hosmer-Lemeshow goodness-of-fit statistic (p > 0.1) supported Model 2, with both distance to the clinic and distance to the road as regression covariates, as the better fitting model. Since the clinic and swamp were very close to each other, distance to the swamp was not considered in the regression analysis.

**Table 2:** Logistic regression results identifying factors associated with higher proportion of RDT positive household residents

	Univariate*		Model 1		Model 2	
	OR (95% CI)	P value	OR (95% CI)	P value	OR (95% CI)	P value
Index is female	1.08 (0.89, 1.31)	0.45	1.15 (0.93, 1.41)	0.18	1.15 (0.93, 1.41)	0.19
Index under 5 years of age	1.42 (1.17, 1.73)	<0.01	1.41 (1.15, 1.73)	<0.01	1.41 (1.15, 1.73)	<0.01
Distance to clinic (per 500 m)	1.03 (1.01, 1.05)	<0.01	–	–	1.00 (0.98, 1.03)	0.44
Distance to road (per 500 m)	1.05 (1.03, 1.07)	<0.01	1.05 (1.03, 1.07)	<0.01	1.05 (1.02, 1.07)	0.01
Rainy season	1.03 (0.84, 1.25)	0.81	1.16 (0.95, 1.43)	0.17	1.17 (0.95, 1.45)	0.14

* Univariate model considers each variable as the only covariate.

## Discussion

Significant spatial and seasonal variation of incident malaria infections that reported to Chinyunyu clinic was identified. The space–time clusters of index households, controlling for distance to the road and to the clinic, suggests that factors other than access to care are impacting the distribution of index malaria cases in this catchment area. Potentially, transmission near the swamp is occurring, causing spatial confounding, since the swamp is located near the clinic and along the road. The identified clusters also occurred during the rainy season, suggesting a high number of cases during this period but perhaps also highlighting that in the rainy season roads wash out, and when access is restricted cases that are reported at the clinic are only from nearby areas.

Incident malaria cases reported in the rainy season clustered significantly more than in the dry season. However, the spatial intensity maps identified no difference in the spatial intensities per season. This suggests that cases were occurring in the same general area, but as demonstrated with the K-function difference method the rainy season the pattern was more spatially compact than in the dry season. The higher level of spatial clustering of incident malaria during the rainy season is likely because houses further from the clinic are unable to travel to the clinic during this time. Future studies should include a measurement of care or treatment-seeking behaviour to apply to the area, to control for the probability of an individual in the area seeking care for malaria in a model designed to measure surveillance.

Index households located farther from the road had a significantly higher number of asymptomatic, RDT positive household contacts that were not identified during passive detection, controlling for season. Additionally, if the index case was under the age of five, the household had more RDT positive household contacts. Likely, infections in younger children experienced more severe symptoms and were brought to the clinic for care and enrolling as index cases. These findings suggest that among index households, distance to the clinic may impact reporting and treatment of household contacts. As distance from the clinic increases, asymptomatic infections are less likely to receive testing and treatment, potentially leading to an increased infectious duration [18]. This may impact ongoing transmission in the area.

The major limitations of this study are that information was not collected at control households, and that only a random selection of clinical cases were enrolled as index cases; both limited analyses. Without control households, it was not possible to determine if the prevalence of asymptomatic infections was higher in passively detected households. However, this has been suggested in several studies [4, 18, 19]. The random selection for enrolment means that many infected persons were not enrolled, however, since cases were randomly selected it can be assumed that misclassification is unbiased. Additionally, the findings may have been spatially confounded. It is unclear if the significant clustering of cases is due to increased risk of transmission from the swamp, or if being closer to the road and clinic results in improved access. Another limitation is that potentially enumeration was incomplete, but with up to date and highly accurate satellite imagery this should not have been the case, as this methodology has previously been shown to be accurate [13].

Future research should examine the efficiency of RACD in treating asymptomatic infections missed by passive surveillance. In a setting such as this clinic, the incidence may be too high to recommend RACD of all incoming cases. Although our study lacked control cases and could not assess how much RACD increases detection of asymptomatic infections, previous studies have found that asymptomatic infections spatially cluster in or near index households, suggesting that RACD targeted to the index household may be useful in identifying and treating remaining infections [4]. Our findings support the suggestion that passive surveillance alone will not be sufficient to eliminate malaria, and complementary systems are necessary. This study found that having an index case under 5 years of age increased the chances of identifying asymptomatic infections in the household. This may be because children under 5 are more likely to experience symptoms and be brought in care, identifying household locations that are at increased risk for malaria transmission. Future studies regarding RACD should account for the underlying transmission intensity and include control households in order to determine key remaining questions regarding RACD including: (1) at what transmission intensity is RACD most efficient and effective for identifying asymptomatic cases, (2) how does RACD compare to active case detection methods such as randomly visiting households, (3) how many additional infections are treated with RACD compared to just passive surveillance.

## Conclusions

These findings suggest that there is significant spatial and seasonal variation of incident (index) cases related to proximity to the clinic and main road. Malaria cases identified from the clinic were reported from the same geographic area, regardless of the season. However, the clusters detected occurred in the rainy season, and spatial clustering was higher in the rainy season, suggesting that when smaller roads wash out, only households closer to the clinic are able to seek care for malaria. Index cases tended to be under 5 years of age and have asymptomatic household contacts, mainly older children that did not seek care. Evaluating the spatial and temporal distribution of households can determine the impact of clinic-based interventions and improve the coverage of treatment and preventive interventions.
